# Coffee Bioactive *N*-Methylpyridinium Attenuates Tumor Necrosis Factor (TNF)-α-Mediated Insulin Resistance and Inflammation in Human Adipocytes

**DOI:** 10.3390/biom11101545

**Published:** 2021-10-19

**Authors:** Stefano Quarta, Egeria Scoditti, Maria Annunziata Carluccio, Nadia Calabriso, Giuseppe Santarpino, Fabrizio Damiano, Luisa Siculella, Martin Wabitsch, Tiziano Verri, Claudia Favari, Daniele Del Rio, Pedro Mena, Raffaele De Caterina, Marika Massaro

**Affiliations:** 1 Department of Biological and Environmental Sciences and Technologies (DISTEBA), University of Salento, 73100 Lecce, Italy; stefano.quarta3@unisalento.it (S.Q.); fabrizio.damiano@unisalento.it (F.D.); luisa.siculella@unisalento.it (L.S.); tiziano.verri@unisalento.it (T.V.); 2 Institute of Clinical Physiology (IFC), National Research Council (CNR), 73100 Lecce, Italy; egeria.scoditti@ifc.cnr.it (E.S.); maria.carluccio@ifc.cnr.it (M.A.C.); nadia.calabriso@ifc.cnr.it (N.C.); 3 Cardiovascular Center, Paracelsus Medical University, 90471 Nuremberg, Germany; gsantarpino@gvmnet.it; 4 GVM Care & Research, Città di Lecce Hospital, 73100 Lecce, Italy; 5 Cardiac Surgery Unit, Department of Experimental and Clinical Medicine, University “Magna Graecia”, 88100 Catanzaro, Italy; 6 Division of Pediatric Endocrinology, Diabetes and Obesity, Department of Pediatrics and Adolescent Medicine, University of Ulm, 89075 Ulm, Germany; Martin.Wabitsch@uniklinik-ulm.de; 7 Human Nutrition Unit, Department of Food and Drugs, University of Parma, 43125 Parma, Italy; claudia.favari@unipr.it (C.F.); daniele.delrio@unipr.it (D.D.R.); pedromiguel.menaparreno@unipr.it (P.M.); 8 Cardiology Division, Pisa University Hospital, 56126 Pisa, Italy; 9 Fondazione Villa Serena per la Ricerca, Città Sant’Angelo, 65013 Pescara, Italy

**Keywords:** coffee bioactives, *N*-methylpyridinium, adipocytes, inflammation, insulin resistance

## Abstract

Although coffee consumption has been historically associated with negative health outcomes, recent evidence suggests a lower risk of metabolic syndrome, obesity and diabetes among regular coffee drinkers. Among the plethora of minor organic compounds assessed as potential mediators of coffee health benefits, trigonelline and its pyrolysis product *N*-methylpyridinium (NMP) were preliminary shown to promote glucose uptake and exert anti-adipogenic properties. Against this background, we aimed at characterizing the effects of trigonelline and NMP in inflamed and dysfunctional human adipocytes. Human Simpson-Golabi-Behmel syndrome (SGBS) adipocytes were treated with NMP or, for comparison, trigonelline, for 5 h before stimulation with tumor necrosis factor (TNF)-α. NMP at concentrations as low as 1 µmol/L reduced the stimulated expression of several pro-inflammatory mediators, including C-C Motif chemokine ligand (CCL)-2, C-X-C Motif chemokine ligand (CXCL)-10, and intercellular adhesion Molecule (ICAM)-1, but left the induction of prostaglandin G/H synthase (PTGS)2, interleukin (IL)-1β, and colony stimulating factor (CSF)1 unaffected. Furthermore, NMP restored the downregulated expression of adiponectin (ADIPOQ). These effects were functionally associated with downregulation of the adhesion of monocytes to inflamed adipocytes. Under the same conditions, NMP also reversed the TNF-α-mediated suppression of insulin-stimulated Ser473 Akt phosphorylation and attenuated the induction of TNF-α-stimulated lipolysis restoring cell fat content. In an attempt to preliminarily explore the underlying mechanisms of its action, we show that NMP restores the expression of the master regulator of adipocyte differentiation peroxisome proliferator-activated receptor (PPAR)γ and downregulates activation of the pro-inflammatory mitogen-activated protein jun N-terminal kinase (JNK). In conclusion, NMP reduces adipose dysfunction in pro-inflammatory activated adipocytes. These data suggest that bioactive NMP in coffee may improve the inflammatory and dysmetabolic milieu associated with obesity.

## 1. Introduction

The seriousness of the global epidemic of obesity and type 2 diabetes (T2D), now collectively referred to as “diabesity”, can no longer be questioned [1]. Updated projections estimate a sixfold increase in the number of adults with obesity in the next 40 years, while the number of individuals with diabetes seems destined to reach 642 million by 2040 [2]. Since “diabesity” significantly increases the risk of serious cardiovascular sequelae [3], the consequent huge health burden has prompted the search for new preventive or therapeutic strategies to relieve metabolic dysfunctions in obese diabetic patients. Despite significant advances in T2D pathophysiology over the last decades [4], the exact mechanisms through which obesity causes T2D are not completely understood. The most credited pathogenic link remains the development of a low-grade inflammatory condition involving, but not limited, to, visceral white adipose tissue (WAT) [5]. Under excessive caloric intake, hypertrophic white adipocytes switch their secretory activity towards a more pro-inflammatory phenotype, activating the production or downregulation of an array of adipose-derived factors, called adipokine [6]. Tumor necrosis factor (TNF)-α is a cytokine secreted by both adipocytes and immune cells within obese WAT [7]. Compared with adipose tissue in lean subjects, TNF-α expression is many times higher in obese subjects [8,9] in correlation with hyperinsulinemia [10]. TNF-α increases the release of lipolysis and free fatty acids (FFA), deregulating the activity and the expression of the complex set of lipases and related cofactors, including the expression of the adipose triglyceride lipase (ATGL), thus promoting and sustaining insulin resistance in the liver and in skeletal muscle [11]. Furthermore, TNF-α inhibits the phosphorylation of insulin receptor substrate (IRS)-1 and serine/threonine protein kinase Akt by activating c-Jun N-terminal kinase (JNK) and IκB kinase (IKK), thereby preventing insulin signal transduction [12]. Finally, in an animal model of diet-induced obesity, inflammation also drove the expression of the leukocyte adhesion molecule intercellular adhesion molecule (ICAM)-1, which accelerated the adipose homing of monocytes, thereby perpetuating both adipose and vascular inflammation [13,14].

Coffee is among the most consumed drinks worldwide, with consumption rates continuously increasing due to renewed consumer perceptions of the health benefits achievable through a regular coffee intake [15]. In prospective cohort studies, long-term consumption of coffee has been consistently associated with a reduced risk of T2D [16] and, to a lesser extent, to reduced adiposity, particularly in men [17]. Interestingly, the association with diabetes risk has been observed with the same efficacy for decaffeinated and caffeinated coffee [18], suggesting that coffee phytochemicals, beyond caffeine, have to be viewed as potential candidates for anti-diabetic effects. Coffee phytochemicals vary, depending on cultivar conditions and manufacturing procedure. In particular, the roasting of coffee beans causes the thermal degradation of trigonelline (TRIGO) and the accumulation of its degradation products, such as *N*-methylpyridinium (NMP) [19], which is known to mediate significant cytoprotective and antioxidant effects [20]. More pertinently to “diabesity”, NMP-rich dark roast coffee administration in humans was shown to reduce body weight [21], enhance thermogenesis, and promote glucose uptake in liver cells [22]. Against this background, since no experimental or mechanistic evidence is available on the direct effects of NMP on hypertrophied inflamed adipocyte, we aimed at characterizing the effects of TRIGO and NMP on inflammation and insulin resistance in dysfunctional human adipocytes, and to explore the underlying mechanisms of action.

## 2. Materials and Methods

### 2.1. Materials

NMP and TRIGO were obtained from Merck (Darmstadt, Germany). Human insulin was obtained from Roche Diagnostics (Mannheim, Germany). Chemical and reagents used to measure proteins and perform electrophoresis were obtained from Bio-Rad Laboratory (Hercules, CA, USA). Unless otherwise indicated, all the other chemicals were from Sigma Aldrich (St. Louis, MO, USA).

### 2.2. Cell Culture and Treatments

Simpson-Golabi-Behmel syndrome (SGBS) preadipocytes were maintained in culture and differentiated into adipocytes, as described in previous research [23]. For the experiments involving stimulation with insulin, the differentiated adipocytes were first transferred to low-glucose DMEM (1000 mg/L) for 24 h and then exposed to NMP for 5 h. Next, some monolayers were exposed to 10 ng/mL TNF-α for 24 h before being stimulated with insulin for 20 min. THP-1 cells, as a model of human monocytic cells, were purchased from the American Tissue Culture Collection (Rockville, MD, USA) and cultured in RPMI 1640 supplemented with 10% FBS, in addition to 2 mmol/L glutamine, 100 mg/mL streptomycin, and 100 IU/mL penicillin, and maintained in a 5% CO_2_-humidified atmosphere at 37 °C. To prevent cell differentiation, the cell density was maintained at less than 1 × 10^6^ cells/mL.

### 2.3. Cell Viability

The cell viability was determined by a 3-(4,5-dimethylthiazol-2-yl)-2,5-diphenyl tetrazolium bromide (MTT) assay, which is a commonly used method to evaluate cell survival, based on the ability of viable cells to convert MTT, a soluble tetrazolium salt, into an insoluble formazan precipitate, which is then quantitated spectrophotometrically. Briefly, after NMP and TRIGO treatment and TNF-α challenge, the cells were incubated with MTT (0.5 mg/mL) for 4 h, and the formazan products were then dissolved by isopropanol. The absorbance was measured at 490 nm by a microplate reader.

### 2.4. Assessment of Cellular Lipid Accumulation

The accumulation of cell lipids was determined by ORO staining. After NMP treatment and TNF-α stimulation, the cells were washed twice with phosphate-buffered saline (PBS) before being fixed in 10% formalin for 30 min and incubated with ORO staining solution for 1 h. After the unbound dye was removed by serial washing with water, the ORO-stained cultures were photographed with an inverted microscope (Leica, Wetzlar, Germany) and the images were captured with an attached Canon Powershot S50 digital camera at a 200× magnification (Carl Zeiss, Oberkochen, Germany). Next, the dye retained in the cells was eluted with isopropanol and finally quantified at 510 nm using a microplate reader. The value of OD510 was considered as an indirect measure of intracellular triglyceride content.

### 2.5. Determination of Lipolysis

Lipolysis was evaluated by the biochemical determination of the amount of glycerol released into the culture media. Briefly, after NMP treatment and TNF-α stimulation for 24 h, the medium aliquots were removed and kept at −20 °C for the subsequent measurement of glycerol concentration (an index of lipolysis) through a direct colorimetric procedure using a commercial kit (Cayman Chemical, Ann Arbor, MI, USA). Briefly, the conditioned medium was incubated with glycerol kinase, glycerol phosphate oxidase, and horseradish peroxidase in the presence of a colorimetric substrate to generate a chromophore, whose intensity was read at 540 nm using a microplate reader. Under these conditions, the amount of glycerol released was correlated with both the amount of stored triglyceride and the degree of subsequent lipolysis.

### 2.6. RNA Isolation and Real-Time Quantitative Polymerase Chain Reaction

The total RNA was isolated by using TRIzol reagent (Invitrogen, now a brand of Thermo Fisher Scientific, Waltham, MA USA), according to the manufacturer’s protocol. The total RNA (2 μg) was used for the cDNA synthesis. A High-Capacity cDNA Reverse Transcription Kit (Applied Biosystems, now a brand of Thermo Fisher Scientific, Waltham, MA, USA) was used to reverse-transcribe RNA to cDNA. The reaction was carried out on a GeneAmp PCR System 9700 (Applied Biosystems) under the following conditions: 10 min at 25 °C, 120 min at 37 °C, and 5 min at 85 °C. Quantitative real-time PCR (qPCR) analyses were performed with the CFX96 Touch Real-Time PCR Detection System instrument and software (Bio-Rad). All the reactions were performed in a total volume of 25 μL containing 50 ng of cDNA, 0.3 pmol/L of a primer pair, and 12.5 μL of the 2× SYBR Green PCR master (Bio-Rad) mix under the following conditions: 2 min at 50 °C, 10 min at 95 °C, and 40 cycles of 15 s at 95 °C and 1 min at 60 °C. The reactions were carried out in triplicate on three independent sets of RNA. Negative controls (no RNA added) were processed under the same conditions as the experimental samples. The amount of mRNA was calculated by using the comparative critical threshold (CT) method. To account for possible variations related to cDNA input or the presence of PCR inhibitors, the endogenous reference gene ribosomal 18 S was simultaneously quantified for each sample and the data were normalized accordingly. The primer sequences used are shown in the Table 1.

### 2.7. Cell Lysis and Immunoblotting

After NMP treatment and TNF-α stimulation, the adipocytes were processed as described in previous research [23]. The obtained blots were incubated overnight at 4 °C with primary antibodies against: p-SAPK/c-Jun N-terminal kinases (JNK) (Thr183/Thr185) (Santa Cruz Biotechnology, Dallas, TX, USA), JNK2 (Merck Millipore Burlington, MA, USA), pAkt (Ser473) (Cell Signaling Technology, Danvers, MA, USA), Akt (Cell Signaling Technology), peroxisome proliferator-activated receptor (PPAR)γ (Santa Cruz Biotechnology, Dallas, TX, USA), and β-actin (Santa Cruz Biotechnology, Dallas, TX, USA), followed by appropriate horseradish peroxidase-conjugated secondary antibodies (Santa Cruz Biotechnology, Dallas, TX, USA). A chemiluminescence kit was used to visualize the protein bands, which were then quantitatively analyzed and normalized to β-actin levels using Scion Image Alpha 4.0.3.2 software (National Institutes of Health, Bethesda, MD, USA).

### 2.8. 2-NBDG Uptake Assays

The uptake of the fluorescent glucose analog 2-[*N*-(7-nitrobenz-2-oxa-1,3-diazol-4-yl) amino]-2-deoxy-d-glucose (2-NBDG) was estimated as described in previous research [23]. In brief, after SGBS differentiation in 24-well plates, the mature adipocytes were transferred to low glucose concentration medium (1000 mg/mL, DMEM) for 24 h before treatment with NMP for 5 h and TNF-α for the following 18 h. After this time, adipocytes were challenged with 100 nmol/L insulin for 30 min. The culture medium was then removed and replaced with PBS containing 0.1% bovine serum albumin with or without 100 µmol/L 2-NBDG for 1 h. After incubation, the free 2-NBDG was washed out from the cultures, and the fluorescence of the cells was recorded with a fluorescence reader. False positives were ruled out by treating the adipocytes as described above, but in the absence of 2-NBDG, and using these measurements as the background. Relative fluorescence intensities normalized to protein content were used for the data analysis.

### 2.9. In Vitro THP-1 Adhesion Assay

The fully differentiated SGBS cells were treated with NMP at the indicated concentrations for 5 h and then stimulated with 10 ng/mL TNF-α for a further 18 h. To examine how many monocytes attached to the cultured adipocytes, the THP-1 cells (10^6^ cells/mL) were fluorescently labelled by incubation with calcein-AM (5 ng/mL) for 30–45 min, and washed twice in an RPMI medium. The suspended THP-1 cells were then added to the SGBS monolayers. After 1 h, the non-adhering cells were removed by gentle washing with DMEM-F12 and monolayers fixed with 1% paraformaldehyde. Images of SGBS and adherent calcein-labeled THP-1 cells were visualized and captured with a stereomicroscope (Nikon, Minato, Tokyo, Japan), equipped with Nikon NIS-Elements D at 40× magnification.

### 2.10. In Silico Molecular Docking

Molecular docking is a computational modelling technique that allows the prediction of molecular interactions that hold together a protein and a ligand in the bound state [24]. The crystal structures of the selected target proteins were derived from Protein Data Bank (PDB, www.wwpdb.org) (accessed on 12 August 2021) with PDB IDs as follows: 5NHV for ERK2, 2H96 for JNK1, 6M9L for p38, and 3A1F for gp91phox. The predicted crystal structures of NOX4 were derived from AlphaFold Protein Structure Database (www.https://alphafold.ebi.ac.uk/) (accessed on 12 August 2021) with the following Uniprot (www.uniprot.org/) (accessed on 12 August 2021) ID: Q9NPH5. In order to prepare the proteins for the docking simulation, all the missing atoms were repaired. In addition, all the water molecules and the co-crystalized heteromolecules were removed, followed by the addition of polar hydrogen atoms and neutralization using Kollman united-atom charges. The dimensions of the grid box were 60 × 60 × 60 with a 0.500 Å distance between the points. During the docking procedure, the ligand was flexible and the protein was rigid. Autodock4 and Lamarckian genetic algorithms were used to dock 250 conformations. The best docked pose was saved and the results of the best poses for the proteins with NMP were analyzed using free energy of binding (ΔG) and the Inhibition Constant (Ki). A Protein-Ligand Interaction Profiler (PLIP, plip-tool.biotec.tu-dresden.de) and Discovery studio 2020 Visualizer were used to investigate the protein-ligand non-bonding interactions of the best poses. The docking calculations were conducted using Autodock Tools 1.5.6.

### 2.11. Statistical Analysis

The results were expressed as means ± S.D. Student’s t-test was used to compare the means between the control group and the compound-treated group. Multiple comparisons were made using one-way analysis of variance (ANOVA). A *p* value of <0.05 was considered statistically significant.

## 3. Results

### 3.1. Effect of NMP on Cell Viability

**Figure 1:**
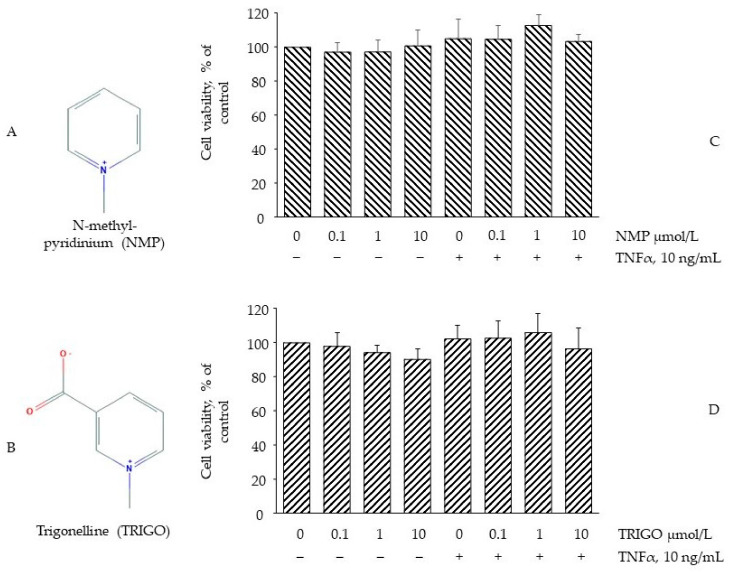
The effect of N-methyl-pyridinium (NMP) and trigonelline (TRIGO) treatment on cell viability. (**A**) Chemical structure of NMP; (**B**) Chemical structure of TRIGO. Adipocytes were treated with NMP (**C**) or TRIGO (**D**) for 5 h at the concentrations indicated, and then either treated with 10 ng/mL TNF-α or left untreated for 18 h. Cell viability was assessed by a 3-(4,5-dimethylthiazol-2-yl)-2,5-diphenyl tetrazolium bromide (MTT) assay, and expressed as a percentage of the unstimulated control. Data (means ± S.D., n = 3) are expressed as percentage of unstimulated control.

The conversion of the water-soluble yellow dye MTT to insoluble purple formazan crystals by the action of mitochondrial reductases (MTT assay) was used to evaluate whether concentrations of NMP or TRIGO (Figure 1A,B), equivalent to those attainable in vivo [25,26], might cause cell damage or toxicity [27]. To this end, fully differentiated SGBS adipocytes were incubated with increasing concentrations of NMP or TRIGO for 5 h before being challenged, or not, with TNF-α for a further 18 h. As shown in Figure 1C,D, the treatment of SGBS cells with NMP or TRIGO up to a concentration of 10 µmol/L did not affect cell viability, either in the absence or in the presence of TNF-α. At the same time, morphological examination under a light-inverted microscope revealed no change in cell morphology or loss of adherence potential.

### 3.2. NMP Restores the TNF-α-Mediated Impairment of GLUT-4 Gene Expression and Insulin-Stimulated Glucose Uptake

**Figure 2:**
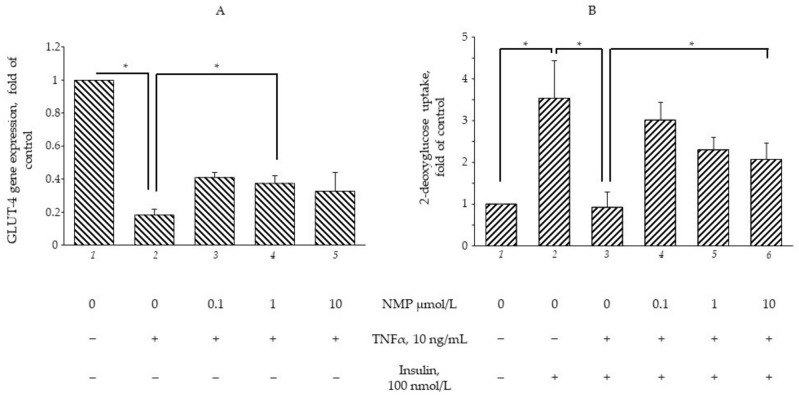
NMP attenuates TNF-α-mediated downregulation of GLUT-4 mRNA expression and improves glucose uptake. (**A**) SGBS adipocytes were pretreated with NMP at the concentrations indicated (5 h) and then treated with 10 ng/mL TNF-α for 18 h. Total RNA was extracted from cells, and mRNA levels of GLUT-4 were measured by qPCR using specific primers and normalized to 18S RNA. Data (means ± S.D., n = 3) are expressed as fold induction over basal (untreated) control, * *p* < 0.05 between groups joined by the horizontal lines. (**B**) SGBS adipocytes were starved for 24 h before treatment with NMP. After 5 h, cells were made insulin-resistant by exposure to TNF-α for 18 h and then stimulated with 100 nmol/L insulin for 1 h. Next, cells were washed and incubated with fluorescent glucose analogue 2-(*N*-(7-nitrobenz-2-oxa-1,3-diazol-4-yl)amino)-2-deoxyglucose (2-NBDG) for 1 h at 37 °C. After washing, fluorescence activity remaining in the cells was measured by a fluorescence microplate reader. Data (means ± S.D., n = 3) are expressed as fold induction over basal (untreated) control. * *p* < 0.05 between groups joined by the horizontal lines.

The consolidated evidence ascribes a pivotal role to TNF-α in orchestrating obesity-related metabolic dysfunction as shown, in vitro, by the reduced uptake of glucose in hypertrophic adipocytes persistently exposed to TNF-α [28]. To evaluate whether NMP and TRIGO were able to alleviate metabolic dysfunction by restoring insulin sensitivity, SGBS adipocytes were exposed to NMP or TRIGO for 5 h and then stimulated with TNF-α for 18 h. As shown in Figure 2A, cell exposure to TNF-α alone significantly reduced GLUT-4 gene expression by 80% (*p* < 0.01). The exposure to NMP, at concentrations of 0.1 and 1 µmol/L, before TNF-α challenge, significantly ameliorated the downregulation of GLUT-4 mRNA (*p* < 0.05), whereas concentrations equal to or above 10 µmol/L, even while improving the downregulating effect exerted by TNF-α, were less effective. Under the same experimental conditions, cell exposure to TRIGO before cytokine-stimulation did not exert any gene-regulating effects (data not shown). For this reason, we decided to perform the following investigations, evaluating only the metabolic activities of NMP. From a functional point of view, the exposure of SGBS adipocytes to insulin increased the glucose uptake by 250% (Figure 2B, bar 2 vs. bar 1, *p* < 0.05). When the cells were exposed to TNF-α for a sufficiently long time (18 h) to induce insulin resistance [28], the insulin-stimulated uptake of glucose was downregulated by 75% (Figure 2B, bar 3 vs. 2, *p* < 0.05). However, when the cells were exposed to NMP before TNF-α challenge, the glucose uptake was significantly restored. Again, lower NMP concentrations exhibited stronger regulating effects (Figure 2B, bar 5 vs. 3, *p* < 0.05).

### 3.3. NMP Improves the Insulin Signaling Impaired by TNF-α

**Figure 3:**
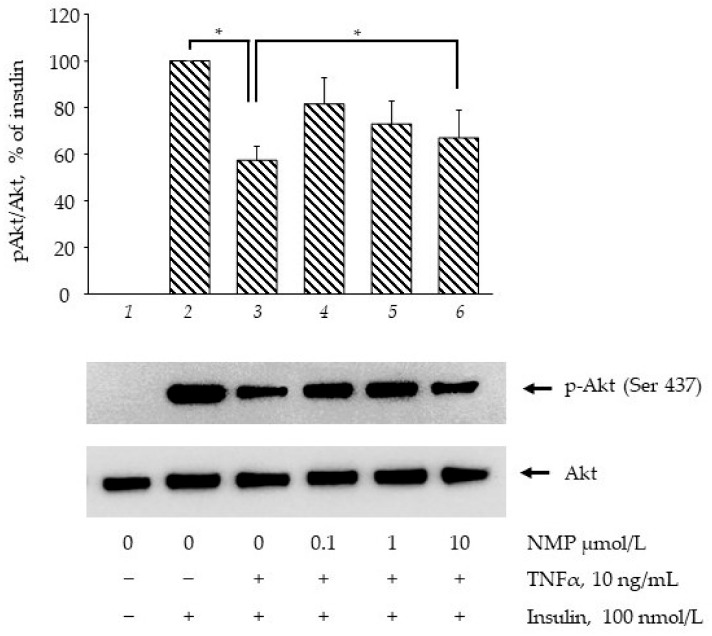
NMP attenuates TNF-α-mediated suppression of insulin signalling. SGBS adipocytes were starved for 24 h before being treated with NMP. After 4 h, cells were made insulin-resistant by exposure to TNF-α for 18 h and then stimulated with 100 nmol/L insulin for 20 min. Whole-cell lysates were assayed by Western blotting using antibodies against phosho(p)Akt and total Akt. Intensity of protein bands were quantified by densitometry and results (means ± S.D., n = 3), expressed as pAkt/Akt, were presented as percentage of insulin. * *p* < 0.05 between groups joined by the horizontal lines.

Ser(S)473Akt phosphorylation (and activation) represents a key event in insulin signaling, and is a commonly estimated target to explore the effects of drugs and bioactives in terms of insulin signalling pathway improvement [29]. As shown in Figure 3, the stimulation of the SGBS adipocytes with 100 nmol/L insulin for 20 min increased S473Akt phosphorylation (bar 2 vs. bar 1, *p* < 0.05). When the cells were made insulin-resistant by exposure to 10 ng/mL of TNF-α for 18 h, the insulin-mediated Akt phosphorylation was decreased by 43% (bar 3 vs. bar 2, *p* < 0.05). However, the cells’ pre-treatment with increased concentrations of NMP significantly restored, by 42%, 27% and 16%, the insulin-mediated AKT phosphorylation (bar 4–6 vs. bar 3, *p* < 0.05). These data showed that NMP, especially at lower concentrations, improves insulin resistance counteracting the TNF-α-induced downregulation of GLUT-4 mRNA, the impairment of insulin-stimulated glucose uptake, and Akt activation.

### 3.4. NMP Modulates Fat Accumulation in Adipocytes Reverting TNF-α Lipolytic Activity

**Figure 4:**
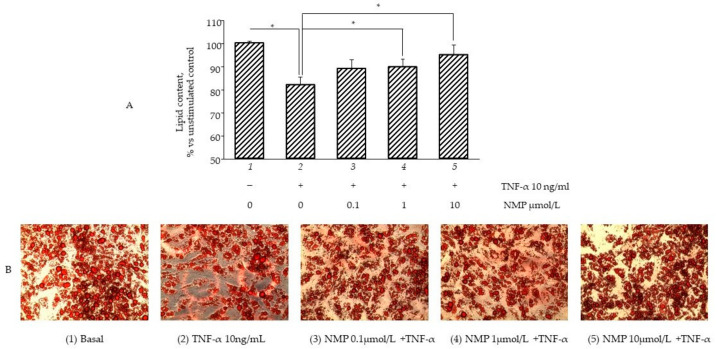
NMP modulates lipid accumulation reverting TNF-α lipolytic activity. Fully differentiated SGBS adipocytes were pretreated with NMP for 5 h and then were treated with 10 ng/mL TNF-α for 3 days. Intracellular lipid accumulation was evaluated by Oil Red O (ORO) staining. (**A**) Spectrophotometric quantification of ORO stain extracted from SGBS adipocytes. (**B**) Microphotographs of ORO staining of SGBS adipocytes. Data (means ± S.D., n = 3) are expressed as percentage over basal (untreated) control. * *p* < 0.05 between groups joined by the horizontal lines.

**Figure 5:**
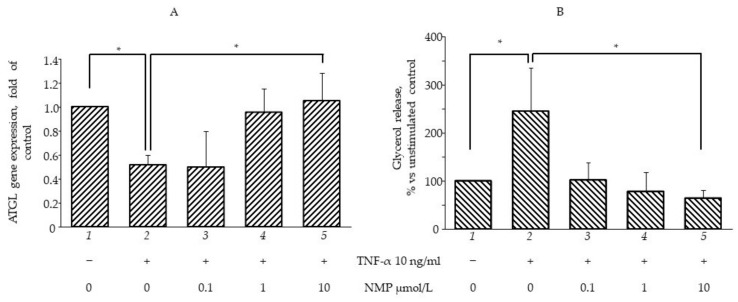
NMP prevents TNF-α induced lipolysis. SGBS adipocytes were pretreated with NMP (4 h) and then treated with 10 ng/mL TNF-α for 18 h. (**A**) Total RNA was extracted from cells, and mRNA levels of ATGL were measured by qPCR using specific primers and normalized to 18S RNA. Data (means ± S.D., n = 3) are expressed as fold induction over basal (untreated) control. (**B**) Media were collected and total amount of released glycerol was measured as an index of lipolysis. Data (means ± S.D., n = 3) are expressed as percent of basal (untreated) control. * *p* < 0.05 between groups joined by the horizontal lines.

It is well known that inflamed insulin-resistant adipocytes are characterized by low lipogenic capacity and high lipolytic activity, which causes increased FFA release [30]. In an attempt to simulate in vitro the pro-inflammatory deregulation of lipid metabolism, we exposed fully differentiated SGBS cells to TNF-α for different times (from 1 to 72 h) before measuring the intracellular lipid content by using ORO staining. In a preliminary set of experiments, we observed that three day-exposure to TNF-α maximally reduced cellular lipid content (data not shown), and therefore we chose this time in the following experiments aimed at evaluating the potential anti-lipolytic activity of NMP. As shown in Figure 4A, cell exposure to TNF-α for 72 h reduced the total intracellular lipid content (bar 2 vs. bar 1, *p* < 0.05), which was also highlighted by the reduced lipid droplet density on the ORO photomicrographs (Figure 4B). Pre-treatment with NMP significantly reversed TNF-α lipolytic activity. We observed that the quantity of lipid droplets increased significantly, in a dose-dependent manner, when the adipocytes were treated with NMP before TNF-α challenge (bar 3–5 vs. bar 2, *p* < 0.05). We next investigated whether the TNF-α-mediated reduction in the lipid content of the cells was due, at least in part, to the deregulation of the expression of the genes associated with lipolysis. We observed that the cells’ exposure to TNF-α reduced the mRNA expression of ATGL) (Figure 5A), as well as the expression of HSL and CGI-58 G0S2 (data not shown). The cells’ pre-treatment with NMP reverted the TNF-α downregulation of ATGL mRNA (Figure 5A, bar 4 and 5 vs. bar 2, *p* < 0.05), leaving unaffected the downregulated mRNA expression of the other lipolytic genes assessed (data not shown). In line with these results, TNF-α per se induced a significant increase in glycerol release (Figure 5B, bar 2 vs. bar 1, *p* < 0.05). Again, the cells’ pre-treatment with 0.1–10 µmol/L NMP prevented the TNF-α-mediated induction of glycerol release (Figure 5B, bar 3–5 vs. bar 2, *p* < 0.05).

### 3.5. NMP Inhibits TNF-α Mediated Inflammatory Gene Expression and Reduces Monocyte Adhesion

**Figure 6:**
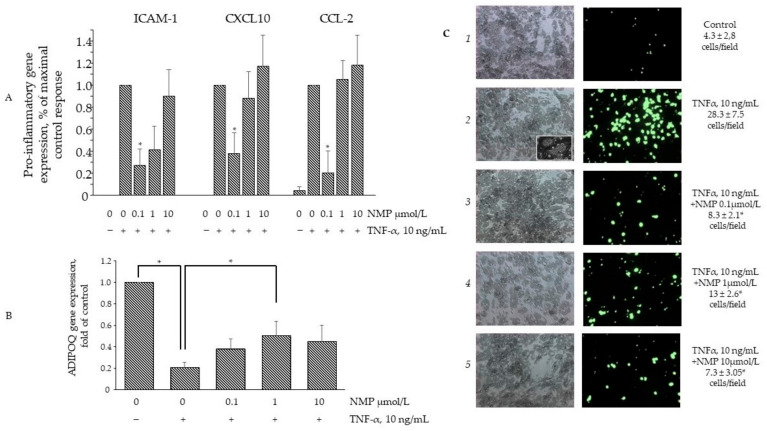
NMP attenuates TNF-α induced adipocytes inflammation. SGBS adipocytes were pretreated with NMP (4 h) and then treated with 10 ng/mL TNF-α for 18 h. (**A**,**B**) Total RNA was extracted from cells, and mRNA levels of ICAM-1, CXCL-10, MCP-1, and ADIPOQ were measured by qPCR using specific primers and normalized to 18S RNA. Data (means ± S.D., n = 3) are expressed as fold induction over basal (untreated) control. * *p* < 0.05 vs. TNF-α alone. (**C**) Fluorescently labeled suspended THP-1 (10^6^ cells/mL) were added to the SGBS monolayers. After 1h, non-adhering cells were removed by three washes and images of SGBS and adherent calcein-labeled THP-1 cells were visualized and captured with a fluorescent microscope at 10× magnification. * *p* < 0.05 vs. TNF-α alone.

Under obesogenic conditions, hypertrophic adipocytes begin to secrete TNF-α, which in turn induces the expression of an array of adipokines able to attract circulating monocytes to the adipose depots, thus further fueling and worsening insulin resistance [30]. The prevention of monocyte attraction and adhesion within the inflamed adipose depots may therefore represent an additional therapeutic target to damper insulin resistance. To this end, we tested the effects of NMP on the expression of a set of adipokines typically deregulated by TNF-α. As expected, TNF-α was able to induce the expression of several pro-inflammatory genes, including CCL-2, CXCL-10, ICAM-1, CSF1. and PTGS2, while it reduced the expression of the anti-inflammatory gene ADIPOQ. As shown in Figure 6A, the exposure of the cells to lower NMP concentrations (equal to 0.1 µmol/L) before TNF-α stimulation was able to counteract the TNF-α-induced expression of the chemokines CCL-2, CXCL-10, and the adhesion molecule ICAM-1 but left the induction of PTGS2, IL-1β, and CSF1 unaffected. Notably, higher NMP concentrations demonstrated no counteracting effects. Furthermore, 0.1 and 1 µmol/L NMP also restored the downregulated expression of ADIPOQ (Figure 6B). We also explored the possible functional consequences of such anti-inflammatory activities by NMP by setting up an adhesion assay between the inflamed adipocytes and the resting monocytes. Due to the global pro-inflammatory reprogramming of the adipocytes, TNF-α powerfully sustained the adhesion of the monocytes to the adipocyte surface (Figure 6C, panel 2 vs. 1; see panel 2 inset for more morphological details of the monocyte adhesion). Under the same experimental conditions, conforming to the NMP anti-inflammatory activity expressed in terms of the adhesion molecules and the chemoattractants, cell exposure to NMP significantly reduced the monocyte adhesion at all the concentrations tested.

### 3.6. NMP Restores the Expression and Activity of Adipogenic and Inflammatory Molecular Switches PPARγ and JNK

**Figure 7:**
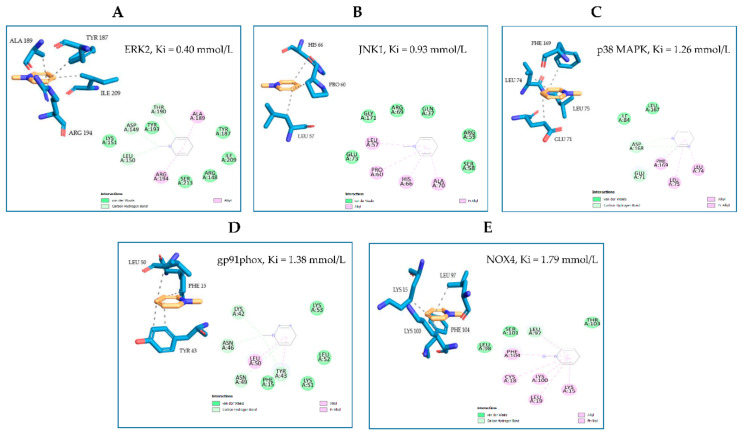
Molecular docking models of putative interactions with target proteins. Putative interactions between NMP and target proteins. (**A**) NMP and ERK1/2 (ERK2; PDB: 5NHV); Free energy of binding (ΔG) and affinity (Ki) are −4.63 kcal/M and 0.400 mmol/L, respectively. (**B**) NMP and JNK (JNK1; PDB: 2H96); ΔG −4.13 kcal/M; Ki 0.93 mmol/L. (**C**) NMP and p38 MAPK (p38MAPK; PDB: 6M9L); ΔG −3.95 kcal/M; Ki 1.26 mmol/L. (**D**) NMP and gp91phox (PDB: 3A1F); ΔG −3.9 kcal/M; Ki 1.38 mmol/L. (**E**) NMP and NOX4 (UNIPROT: Q9NPH5); ΔG −3.75 kcal/M; Ki 1.79 mmol/L.

**Figure 8:**
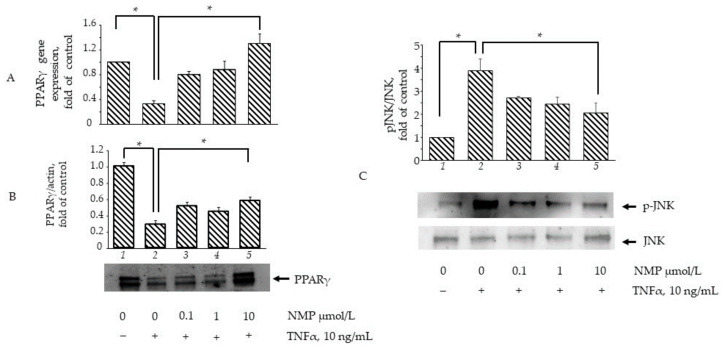
NMP attenuates TNF-α induced inhibition of PPARγ expression and activation of JNK. (**A**) SGBS adipocytes were pretreated with NMP (4 h) and then treated with 10 ng/mL TNF-α for 18 h. Total RNA was extracted from cells, and mRNA levels of PPARγ were measured by qPCR using specific primers and normalized to 18S RNA. Data (means ± S.D., n = 3) are expressed as fold induction over basal (untreated) control. * *p* < 0.05 between groups joined by the horizontal lines (**B**) SGBS adipocytes were pretreated with NMP (4 h) and then treated with 10 ng/mL TNF-α for 18 h. Whole-cell lysates were assayed by Western blotting using antibodies against PPARγ1 and PPARγ2, and against β-actin as a loading control. Intensity of protein bands was quantified by densitometry, and total PPARγ1 and PPARγ2 band intensities were normalized to β-actin (means ± S.D., n = 3) and presented as fold of basal (untreated) control. * *p* < 0.05 between groups joined by the horizontal lines. (**C**) SGBS adipocytes were pretreated with NMP (5 h) and then treated with 10 ng/mL TNF-α for 30 min. Whole-cell lysates were assayed by Western blotting using antibodies against pJNK and total JNK. Intensity of protein bands was quantified by densitometry; results (means ± S.D., n = 3) were expressed as pJNK/JNK, and presented as fold of basal (untreated) control. * *p* < 0.05 between groups joined by the horizontal lines.

In an attempt to elucidate the molecular mechanisms through which NMP affects adipocyte physiology under adipogenic pro-inflammatory conditions, we preliminarily investigated the ability of NMP to interact with key molecular switches in adipocyte biology by performing a predictive computational analysis using a molecular docking approach [24]. To this end, we examined the ability of NMP to interact with representative examples of MAPKs, including JNK, ERK1/2, and p38 MAPK, as well as with gp91phox and NOX4 as representative examples of the catalytic subunits of nicotinamide adenine dinucleotide phosphate (NADPH) oxidase, a multicomponent enzyme also involved in the adipose cellular production of reactive oxygen species (ROS) [31]. The binding affinity, evaluated by ΔG and Ki, non-covalent interactions and hydrophobic interactions, were predicted computationally, and the results are shown in Figure 7. The virtual screening of NMP binding activity indicated that NMP can interact, by non-covalent interactions, with all the target proteins tested. In particular, for the MAPKs tested, the Ki values indicated better affinity for JNK (Ki = 0.93 mmol/L) and ERK2 (Ki = 0.40 mmol/L) than for p38 MAPK (Ki = 1.26 mmol/L). Similar results were obtained when the interaction of NMP with gp91phox (Ki = 1.38 mmol/L) and NOX4 (Ki = 1.79 mmol/L) was evaluated. Considering the key role that JNK plays in orchestrating T2D development [32], we continued our search by analyzing the effect of NMP on the cellular activation of JNK and its downstream effector, PPARγ. We observed that NMP significantly reverted the TNF-α-induced downregulation of PPARγ mRNA (Figure 8A) and, correspondently, reverted TNF-α-induced reduction of PPARγ at protein levels (Figure 8B). At the same time, as shown in Figure 8C, JNK phosphorylation, an index of JNK activity, was induced by TNF-α (Figure 8C, lane 2 vs. lane 1). The cells’ treatment with NMP before TNF-α stimulation significantly reduced JNK activation in a concentration-dependent manner.

## 4. Discussion

Under persistent obesogenic conditions, hypertrophic adipose cells prime local and systemic low-grade inflammatory states that may favor the development of preclinical metabolic dysfunction into overt T2D and related cardiovascular sequelae, especially in centrally obese subjects [5]. In close correspondence with the inflammatory stigmata taking place in atherosclerosis [33], observational and experimental evidence has demonstrated increased monocyte/macrophage infiltration in the hypertrophied adipose tissue [34]. This results in deep systemic metabolic derangements, including “metaflammation” [35] and the dysregulation of plasma lipid profiles, that translate into insulin resistance in more peripheral metabolic tissues, such as the liver and skeletal muscle [36]. Against this background, the dynamic interplay between macrophages and adipocytes is currently viewed as the key process underlying the pathophysiology of insulin resistance and T2D [35]. Therefore, the search for novel drugs or nutraceuticals/bioactive molecules to counteract obesity-driven insulin resistance should target both the adipose pro-inflammatory phenotypic shift and the subsequent systemic metabolic deregulations.

Due to its neuromodulatory effects and its soft bitter taste, the consumption of coffee beverages has significantly increased over the last 150 years, to the point where it has become one of the most traded commodities in the world [15]. Although coffee intake has been historically associated with negative health outcomes, mostly in terms of blood pressure control [37], many more recent studies have highlighted its potential health benefits. Coffee consumption is now associated with a lower risk of metabolic syndrome, obesity, and diabetes [38], and its plethora of minor organic compounds have been assessed as potential sources of nutraceuticals [39]. Coffee plants belong to the Rubiaceae family, which comprises more than ninety species. However, the most commonly cultivated species are the Coffea arabica and the Coffea canephora, which together account for more than 90% of all the world’s coffee production [40].Coffee beans contain polysaccharides, lipids, and proteins as major constituents, as well as a series of minor components, including chlorogenic acids (p-coumaroylquinic, feruloylquinic and caffeoylquinic acids, aka chlorogenic acids), tocopherols, diterpene alcohols (kahweol and cafestol), and two kinds of alkaloids: purine alkaloids, including caffeine (1,3,7-trimethylxanthine) and theobromine (3,7-dimethylxanthine); and pyridine alkaloid TRIGO (1-N-methylnicotinic acid) [41]. Among the coffee components that have attracted the most scientific interest in the last two decades are the alkaloid TRIGO and its thermal transformation products [42]. In an animal model of T2D it was observed that administration of TRIGO prevents diabetes-related organ damage [43], including diabetic nephropathy [44], which suggested TRIGO as a promising bioactive molecule for the treatment of hyperglycemia and metabolic dysfunctions. However, during the roasting process of coffee beans, 50–80% of TRIGO decomposes into nicotinic acid and aromatic nitrogen compounds, including pyrroles, bicyclic compounds, and NMP [45]. Recently, NMP has revealed interesting health benefits, including antioxidant [46] and vasoprotective properties [25], and insulin sensitizing effects in hepatic cell culture [22].

### 4.1. NMP Demonstrated Biological Effects

Here, we report that, at concentrations equivalent to those attainable in vivo [25,26] after the ingestion of 350 mL of freshly prepared dark coffee brew [25] or 1 to 3 espresso coffees [26], NMP exerts anti-inflammatory effects and prevents insulin resistance in human adipocytes made dysfunctional by prolonged exposure to the pro-inflammatory adipokine TNF-α. TNF-α has been proposed as one of the primary humoral links between obesity and insulin resistance [47,48]. Among the thousands of genes deregulated in insulin resistance, TNF-α is among the top 10 hub genes [49], which confirms, for this adipokine, a prominent role in activating and orchestrating signaling pathways that critically translate into the impairment of glucose uptake and the stimulation of lipolysis [49]. For this reason, screening for new bioactives able to interfere with TNF-α signaling assumes strong scientific and clinical relevance. We tested the ability of NMP to interfere with TNF-α signaling in the SGBS cell strain, an adipose cell model that closely resembles human adipocytes [50]. SGBS cells feature a high capacity for adipogenic differentiation and were shown to exert all fat cell-specific metabolic functions, such as insulin-stimulated glucose uptake, insulin-stimulated de novo lipogenesis and β-adrenergic-stimulated lipolysis, as well as secreting typical adipokines, including adiponectin [50]. Under experimental conditions that resembled human adipose insulin resistance (differentiated SGBS made dysfunctional by TNF-α exposure), we observed that NMP: (1) selectively restored the expression of metabolic genes impaired by TNF-α; (2) selectively reversed inflammatory changes in gene expressions induced by TNF-α; (3) attenuated TNF-α-stimulated lipolysis and restored cell fat content; and (4) significantly downregulated the adhesion of monocytes to inflamed adipocytes.

To tentatively explore the underlying mechanisms of action, we showed that NMP reversed the inhibition of S473Akt and restored the expression of the master regulator of adipocyte differentiation and metabolism, PPARγ, possibly by downregulating the activation of JNK. The metabolic regulating effects of other coffee components, including caffeine, caffeic, clorogenic acids [51,52], and kahweol [53] have recently been tested under basal, non-inflamed conditions. Clorogenic and caffeic acids, as well as kahweol, have shown the ability to reduce lipid content [52,53], increase lipolysis [52], improve glucose uptake [52,53], and reduce adipocyte basal inflammation [51]. More pertinently to our experimental setting, Rebollo-Hernanz et al. [54] reported the ability of clorogenic and caffeic acid at supraphysiological concentrations (100 μmol/L), as well as protocatechuic and gallic acids and kaempferol, to inhibit the release of proinflammatory cytokines, such as TNF-α and CCL2, and to counter-regulate the inhibition of anti-inflammatory ADIPOQ under pro-inflammatory conditions. Under obesogenic conditions, inflammatory cytokines induce lipolysis in adipocytes and the consequent release of FFA amplifies inflammatory signalling in both stromal and adipose parenchyma, creating an inflammatory paracrine loop [55]. In close correspondence with our results, clorogenic and caffeic acid, as well as protocatechuic and gallic acids and kaempferol, were shown to prevent the depletion of lipid content and the stimulation of glycerol release [54].

### 4.2. Role of TNF in Insulin Signaling and Lipogenesis

In physiological conditions, insulin’s interaction with its receptor (IR) determines the autophosphorylation of its tyrosine residues that enable IR to attract and activate the IR substrate (IRS)-1, which, in turn, leads to the recruitment and activation of the catalytic subunit of PI3K. Once activated, PI3K converts phosphatidylinositol-4,5-bisphosphate (PIP2) to phosphatidylinositol-3,4,5-triphosphate (PIP3) within the plasma membrane. The interaction of Akt with PIP3 makes possible the activation of Akt by upstream kinases: the 3-phosphoinositide-dependent protein kinase-1 (PDK1) that phosphorylates Akt in Thr 308 (catalytic domain), and the mammalian target of rapamycin complex 2 (mTORC2), DNA-dependent protein kinase (DNA-PK), and ataxia telangiectasia mutated kinase (ATM). This induces the full activity of Akt, phosphorylating it in Ser473 (regulatory domain) [56]. Upon activation, Akt moves to the cytoplasm, where it regulates the activity of a series of target proteins involved in the glycogen synthesis, gluconeogenesis, and lipogenesis while also allowing GLUT-4′s translocation from the intracellular compartment to the plasma membrane via PKCλ/ζ [29]. Finally, Akt is also involved in de novo lipogenesis by regulating a subset of lipogenic genes, including the sterol regulatory element-binding transcription factor 1 (SREBP1c) and PPARγ [57]. Several cell and animal studies showed that TNF-α promotes insulin resistance, inducing the activity of the mitogen-activated protein kinases (MAPKs) ERK1/2 and JNK [58], as well as the activity of the inhibitory kappa B kinase (IKKβ), PKC, mTORC1 and its downstream effector, ribosomal protein S6 kinase (S6K), which, in turn, phosphorylates IRS at Ser307, thereby determining the interruption of insulin signalling [59]. Furthermore, the interaction of TNF-α with its receptor, TNFR1, was shown to upregulate the expression of protein phosphatase 2C (PP2C), leading to the suppression of insulin sensitivity through the inactivation of the energy sensor AMP-activated protein kinase (AMPK) [60]. TNF-α was also shown to modulate the global gene expression profile of adipocytes through the activation of the pro-inflammatory transcription factor Nuclear Factor (NF)-κB [28]. However, although it is well known that the prolonged exposure of adipose cells to TNF-α determines the downregulation of both the transcript and the protein levels of GLUT-4 [28], the molecular mechanisms underlying this effect have only recently begun to be clarified in more detail. In rat muscle cells, it has been observed that TNF-α, by the activation of JNK and ERK1/2 and following NF-κB, induces the upregulation of the co-activator PRIP interacting protein with methyl transferase domain (PIMT), which reduces GLUT-4 by affecting the expression of its transcription factor, Myocyte Enhancer Factor (MEF)2A [61].

### 4.3. Sites of NMP Interference within TNF-α Signaling

We observed that NMP was able to counter-regulate both the TNF-α-mediated downregulated expression of GLUT-4 and the inhibition of S473Akt phosphorylation, suggesting the potential ability of NMP to interfere with mechanisms upstream of IKK activation. In agreement with Ruan et al. [28], we also observed that adipocyte exposure to TNF-α induces a deep gene-reprogramming process that includes the downregulation of PPARγ. PPARγ is a ligand-dependent transcription factor belonging to the steroid receptor superfamily [62,63]. It is highly expressed in adipocytes, and it plays a major role as an regulator of adipogenesis and lipid storage, thermogenesis, and insulin sensitivity [64]. Upon its activation by interaction with natural and synthetic agonists, PPARγ modifies the adipocyte phenotype, upregulating the expression of genes involved in fatty acid metabolism and triglyceride storage, as well as modulating the expression and secretion of a range of factors, including pro- and anti-inflammatory molecules, finely tuning the maintenance of mature adipocyte homeostasis [65]. In agreement with Kim et al. [66], we also observed that adipocyte exposure to TNF-α, as well as downregulating the expression of PPARγ, also downregulates the expression of a series of PPARγ target genes including the anti-inflammatory and insulin sensitizing factor ADIPOQ [67], and the expression of the lipolysis gene set ATGL, HSL, and G0S2 [67,68]. Generally, the directionality of gene expressions regulated by PPARγ leads to and promotes adipose triglyceride storage and/or fatty acid oxidation [69]. Although the PPARγ-mediated induction of ATGL may represent a potential mechanism to increase the supply of fatty acids for oxidation in brown adipocytes, the reason why PPARγ would induce a gene critical to lipolysis in white adipocytes appears less clear. It is possible that increased ATGL expression may reflect a PPARγ-mediated increase in preadipocyte differentiation, as well as a comprehensive effect of PPARγ in maintaining the expression of adipocyte-specific genes in mature adipocytes [70]. In line with the largely invoked anti-inflammatory role of PPARγ activity [71], a new potential anti-inflammatory role has also been demonstrated for its, target gene ATGL [72]. It was recently observed in mature adipocytes that fatty acids released by ATGL activities act as “specific lipid signaling molecules”, leading to the activation of PPARα, which, together with PPARγ, represses the expression of inflammatory genes interfering with NF-κB activity [73]. Correspondently, ATGL deficiency in both visceral adipose tissue and adipocytes was shown to impair PPARα anti-inflammatory signaling [74]. Of note, the well-known bioactive resveratrol has been shown to inhibit the production of pro-inflammatory cytokines in adipocytes upregulating ATGL expression [74]. Under pro-inflammatory conditions leading to the downregulation of ATGL mRNA, our data also demonstrate the ability of NMP to upregulate ATGL, in this way contributing to the global restoration of adipocyte physiology impaired by TNF-α exposure. Several in vitro studies have demonstrated that TNF-α inhibits the transcriptional activity and expression of PPARγ through a mechanism involving the activation of JNK [75]. Correspondingly, in an animal model of obesity, JNK was highly activated in visceral adipose deposits, muscles, and the liver [75]. Moreover, biopsies of the subcutaneous adipose tissue of obese individuals also showed an increased activation of phosphorylated JNK [11,76]. The emerging role of JNK at the intersection between IRS inactivation and PPARγ downregulation suggests that JNK could be a potential key target in the search of new medicines to control insulin resistance and T2D [32]. In obesity, the rapid growth of adipose tissue determines the reduction in the ability of blood vessels to adequately perfuse the whole tissue. This, together with the increased oxygen consumption in the enlarging adipocytes, may result in the establishment of dangerous hypoxic conditions [77]. Cellular hypoxia can trigger inflammation by inducing the hypoxia-inducible factor (HIF)1A gene and endoplasmic reticulum stress, both of which lead to MAPK activation [78]. In the same way, caloric overload also contributes to mitochondrial dysfunction and increases the production of ROS, which also triggers MAPK activation, including JNK and p38 MAPK [79]. Evidence of increased oxidative stress is typically seen in the plasma and in the white adipose tissue of obese mice [80], in which the administration of ROS inhibitors improves hyperglycemia, hyperinsulinemia, and hyperlipidemia and reduces both ROS production and TNF-α expression [80]. Correspondently, in adipocyte cultures, TNF-α-mediated insulin resistance is associates with increased ROS production and JNK activation [81]. The quenching of ROS overload may therefore represent a way to dampen insulin resistance and inflammation. Interestingly, NMP was shown to express antioxidant capacities in in vitro and in vivo models of disease [46]. We hypothesize that in our experimental conditions, NMP acts as an ROS quencher. However, this hypothesis was not directly tested and represents an objective for future experimental evaluation.

## 5. Conclusions

We demonstrated, for the first time, that adipocyte exposure to NMP effectively improves insulin signaling while reducing monocytoid cell adhesion and related stigmata of inflammation, thus further allowing its qualification as an anti-inflammatory molecule. Moreover, taking all these findings together, we propose for NMP the potential to act as an anti-inflammatory and insulin-sensitizing bioactive molecule. This may explain and sustain, at least in part, the anti-diabetic and anti-obesity properties of coffee beverages [16,17]. A limitation of our study is that the findings presented were exclusively obtained using human cell culture systems. Therefore, they have to be regarded as helpful in providing mechanistic insight into NMP metabolic effects, but cannot necessarily be extrapolated to patients, as only findings from clinical trials can provide this level of evidence. It follows that there is an obvious need to further characterize and understand such properties to translate them into clinically safe nutraceuticals or dietary recommendations. Our data, clarifying and confirming the protective anti-inflammatory and insulin-sensitizing mechanisms of NMP, suggests the need for and the potential utility of further testing this molecule in pre-clinical animal models of obesity and diabetes.

## Figures and Tables

**Table 1:** Primer sequences used for qPCR analysis.

Gene Symbol	Full Name	Forward Primer (5′-3′)	Revers Primer (3′-5′)	Accession Number
MCP-1/CCL-2	monocyte chemoattractant protein-1/C-C Motif chemokine ligand 2	CCCCAGTCACCTGCTGTTAT	TCCTGAACCCACTTCTGCTT	NM_002982.3
CXC-L10	C-X-C Motif chemokine ligand 10	CAAGGATGGACCACACAGAG	GCAGGGTCAGAACATCCACT	NM_001565.2
PTGS2/COX-2	prostaglandin G/H synthase 2/cyclooxygenase-2	TGCTGTGGAGCTGTATCCTG	GAAACCCACTTCTCCACCA	NM_000963.2
IL-1β	interleukin 1 β	CTGTCCTGCGTGTTGAAAGA	AGTTATATCCTGGCCGCCTT	NM_000576.2
CSF1/M-CSF	colony stimulating factor 1/macrophage-colony stimulating factor	TGGACGCACAGAACAGTCTC	CCTCCAGGGCTCACAATAAA	NM_000757.4
ADIPOQ	adiponectin	AGTCTCACATCTGGTTGGGG	CTCTCTGTGCCTCTGGTTCC	NM_001177800.1
Slc2a4/GLUT-4	solute carrier family 2 member 4/glucose transporter type 4	TGGTCCATGTACCCCTCATT	AGAGCCTGTGTGGCAAGAGT	NM_009204.2
PPARγ	peroxisome proliferator-activated receptor γ	TGCAGGTGATCAAGAAGACG	AGTGCAACTGGAAGAAGGGA	NM_005037.5
ATGL/PNPLA2	adipose triglyceride lipase/patatin-like phospholipase domain-containing protein 2	CTGACCACCCTCTCCAACAT	TCACCAGGTACTGGCAGATG	NM_020376.4
LIPE/HSL	lipase E, hormone sensitive type/hormone-sensitive lipase	TTCCTCCGGGAGTATGTCAC	TGTGATCCGCTCAAACTCAG	NM_005357
CGI-58	comparative gene identification-58	GCACCCTGACATTCCAGTTT	CAGTCCACAGTGTCGCAGAT	AF151816.1
G0S2	G0/G1 switch gene 2	GGAAGATGGTGAAGCTGTACG	CTTGCTTCTGGAGAGCCTGT	NM_015714
PLIN1	perilipin 1	TCTCGATACACCGTGCAGAC	TGGTCCTCATGATCCTCCTC	NM_002666.5
ICAM-1	intercellular adhesion molecule-1	AGACATAGCCCCACCATGAG	CAAGGGTTGGGGTCAGTAGA	NM_000201.2
18S	18 ribosomal RNA	AAACGGCTACCACATCCAAG	CCTCCAATGGATCCTCGTTA	NR_003286.2

## Data Availability

Not applicable.
